# Supplementary material for: A qualitative exploration of the over-the-counter availability of oral contraceptive pills in Australia
Source: PLoS One. 2024 Jun 10;19(6):e0305085. doi: 10.1371/journal.pone.0305085 (PMC11164330; doi:10.1371/journal.pone.0305085)
Supplement: S1 Appendix — (DOCX) [file pone.0305085.s001.docx]

**Title: A qualitative exploration of the over-the-counter availability of oral contraceptive pills in Australia**

**S1 appendix: COREQ checklist**

| Topic | Item no. | Guide question/description | Answer |
| --- | --- | --- | --- |
| Domain 1: Research team and flexibility | | | |
| Personal characteristics  Interviewer/facilitator | 1 | Which author(s) conducted the interview or focus group? | NG acted as the moderator in phase 1 and ZA led Phase 2. |
| Credentials | 2 | What were the researcher’s credentials? | ZA is a PhD candidate and all other researchers have a PhD as their highest academic qualification |
| Occupation | 3 | What was the researcher’s occupation at the time of the study? | ZA was a PhD researcher and all authors were university researchers, including three associate professors, two Honorary Research Fellows. |
| Gender | 4 | Was the researcher male or female? | The research team constituted five cisgender women aged between 28-45 years and one cisgender man of mixed ethnicity from Australia, New Zealand, India, Bangladesh, China and Zimbabwe living in Australia. |
| Experience and training | 5 | What experience or training did the researcher have? | ZA attended the training event “Credible Qualitative Research- A Masterclass with Professor David Silverman”. She also received a training on NVivo software for qualitative data analysis.  BP and MM attended training on "Qualitative methods for Health Economics " in 2019 with Professor Joanna Coast.  NG and MM have previous experience in conducting FGDs.  YG has extensive experience in preference elicitation studies in health economics involving mixed methods research. |
| Relationship established | 6 | Was a relationship established prior to study commencement? | Yes, BP, KS and YG have ongoing research collaborations. BP, NG and MM  have successfully collaborated on research previously.  YG and MM also have ongoing research collaborations. |
| Participant knowledge of  the interviewer | 7 | Was a relationship established prior to study  commencement? | The participants did not know the researchers prior to the study commencement. At the beginning of the focus groups, the two researchers introduced themselves, including brief information about their research interests and teaching responsibilities. The main objectives of the research project were also explained. |
| Interviewer characteristics | 8 | What characteristics were reported about the  interviewer/facilitator, such as bias,  assumptions, reasons and interests in the  research topic? | See the previous response. The main objective of the research project was also explained at the outset |
| Domain 2: Study design | | | |
| Theoretical framework  Methodological orientation  and theory | 9 | What methodological orientation was stated  to underpin the study, such as grounded  theory, discourse analysis, ethnography,  phenomenology and content analysis? | The focus group data were analysed using thematic analysis – key themes and codes were identified inductively from the transcripts. |
| Participant selection  Sampling | 10 | How were participants selected, for example,  using purposive, convenience, consecutive or  snowball sampling? | Convenience sampling was used for this study. To be eligible, women had to speak English, be between the ages of 18 and 45 and currently not trying to conceive. |
| Method of approach | 11 | How were participants approached? e.g. face-to-face, telephone, mail, email | Women were recruited using an advertisement in the Macquarie University newsletter, and posters displayed in the local vicinity (Macquarie University hospital, University bathrooms and the local shopping centre). |
| Sample size | 12 | How many participants were in the study? | The FGD1a and FGD1b each comprised of 7 women. The second phase of FGD (FGD 2) comprised of 8 women. In three FGDs, 22 women participated in total. |
| Non-participation | 13 | How many people refused to participate or dropped out? Reasons? | Two participants could not participate after expressing their interest, because they were unable to participate in person. |
| Setting | | | |
| Setting of data collection | 14 | Where was the data collected? e.g. home, clinic, workplace | The FGDs were convened at Macquarie University each lasting approximately 90 minutes. To maximize the possibility of the group feeling supportive and open, the FGDs were appropriately facilitated consisting of only female researchers so that the participants were comfortable discussing sensitive contraception issues. |
| Presence of non-participants | 15 | Was anyone else present besides the participants and researchers? | Nobody was present besides the participants and researchers and research assistants named in the acknowledgements. |
| Description of sample | 16 | What are the important characteristics of the sample? e.g. demographic data, date | Described in Table 1 of the manuscript. |
| Data collection | | | |
| Interview guide | 17 | Were questions, prompts, guides provided by the authors? Was it pilot tested? | Questions, prompts and focus group guides were collaboratively developed by all the authors. Although these elements were not specifically pilot tested, they were extensively reviewed by the authors. They also drew on prior qualitative work the authors had been involved in an extensive literature review. |
| Repeat interviews | 18 | Were repeat interviews carried out? If yes, how many? | No, we only ran focus groups |
| Audio/visual recording | 19 | Did the research use audio or visual recording to collect the data? | All FGDs were audio-recorded with the permission of the participants. The FGDs were recorded and transcribed verbatim, and participants identifying information was redacted. Discussions were transcribed using a transcribing service. Participants were contacted again where necessary for clarifying their statements in the transcripts. |
| Field notes | 20 | Were field notes made during and/or after the interview or focus group? | Field notes were taken during the focus group discussions |
| Duration | 21 | What was the duration of the interviews or focus group? | Approximately 90 minutes per focus group discussion |
| Data saturation | 22 | Was data saturation discussed? | We conducted three focus group sessions in two phases. Empirical evidence demonstrates that two to three focus groups likely identify at least 80 per cent of a topic’s themes (Guest et al., 2017). No new code emerged in the FGD 2 regarding the topic, which indicated data saturation. |
| Transcripts returned | 23 | Were transcripts returned to participants for comment and/or correction? | Transcripts were returned to two participants to correct the unintelligible statements. |
| Domain 3: Analysis and findings | | | |
| Data analysis | | | |
| Number of data coders | 24 | How many data coders coded the data? | To aid rigour within the study, the transcripts were coded by both BP and ZA and differences between coding were discussed. |
| Description of the coding tree | 25 | Did authors provide a description of the coding tree? | Yes, in online appendix 3. |
| Derivation of themes | 26 | Were themes identified in advance or derived from the data? | Themes were derived from the data. |
| Software | 27 | What software, if applicable, was used to manage the data? | NVivo-20 was used for analyzing the data. |
| Participant checking | 28 | Did participants provide feedback on the findings? | No, but some participants provided additional information after the focus group sessions. |
| Reporting | | | |
| Quotations presented | 29 | Were participant quotations presented to illustrate the themes/findings? Was each quotation identified? e.g. participant number | Yes. Quotations were identified in a way such that participants’ anonymity was retained. |
| Data and findings consistent | 30 | Was there consistency between the data presented and the findings? | Yes, two researchers analysed the focus group data and all four researchers agreed on the findings. |
| Clarity of major themes | 31 | Were major themes clearly presented in the findings? | Yes, major themes are the headings for the discussion section of the paper. |
| Clarity of minor themes | 32 | Is there a description of diverse cases or a discussion of minor themes? | The analysis and findings discuss divergence among the focus group participants on particular themes, such as the impact of OTC OCP on the use of LARC, utilization of checklist and so on. |

Guest, G., Namey, E., & McKenna, K. (2017). How Many Focus Groups Are Enough? Building an Evidence Base for Nonprobability Sample Sizes. *Field Methods*, *29*(1), 3-22. <https://doi.org/10.1177/1525822X16639015>
