# Supplementary material for: A qualitative exploration of the over-the-counter availability of oral contraceptive pills in Australia
Source: PLoS One. 2024 Jun 10;19(6):e0305085. doi: 10.1371/journal.pone.0305085 (PMC11164330; doi:10.1371/journal.pone.0305085)
Supplement: S2 Appendix — (DOCX) [file pone.0305085.s002.docx]

**Title: A qualitative exploration of the over-the-counter availability of oral contraceptive pills in Australia**

S2 Appendix: Major and minor themes with responses from the participants

| Theme 1: Accessibility and convenience if OCPs are available over the counter | |
| --- | --- |
| Minor themes | Sample response from the participants |
| - Increase access for the younger women | Yeah, or like you said, are still dependent on their parents for Medicare and stuff. They should have access to contraceptives when they want, whichever the want. |
| - Beneficial for low income women | I think it would increase, particularly for like low socio-economic areas where they might not have easier access to doctors. I mean, I think it's really easy to forget being in a certain privileged [unclear] that I remember a few years ago when I found out I was talking to a friend of mine's mum who's a counsellor, and she was counselling these teenage girls who didn’t understand what sanitary products were or how to use them. I couldn’t fathom in my head that that was possible in Australia. But it is and so if you can make things easier and people have an easier access to talk to someone about it, I think you'd be able to get a lot more information out there than probably currently is. |
| - Increase accessibility in terms of opening hours | I just think sometimes you can't always get a doctor's appointment or it's really difficult with work and work hours and so I wish my doctor would let us just call. But it can take like a good week sometimes just to sort it out |
| - Saves time | Time saving, it's just convenient. |
| - Convenient | I think that goes into the convenience part. Like if you were busy and you were really juggling a lot of work and family responsibilities, sometimes it is, you know, OK, I will go tomorrow. OK, maybe not day after tomorrow. So it's kind of a headache like when you have to go several times to the GP. So that's one thing. And accessibility as in like for the people who are having difficulty to reach, like younger people. And I don't know if it will really help people who are like from challenging family circumstances. Like when, you know, people are not supported and you just can't tell, it still – they will get the access if it's easily available obviously. Yes. |
| Theme 2: Cost | |
| - OCPs are expensive | It's also quite, I guess, I don't really have much experience with others, but it's quite expensive, I find, like depending which pill you're on obviously, but I find it is quite expensive. |
| - Non-prescription OCPs saves cost | Yes, I think so. I think more GPs are moving away from bulk billing. So you know, if you have a GP that doesn't bulk bill, you'll get in a repeat prescription, let's say. It's an expense. If it's the first, again, the first time. Maybe it's a suitable model. If it's not, again, that's another hurdle, if you're paying 70 dollars to see your GP. |
| - Pharmacists will have to be paid for their time for Non-prescription OCPs | Yes, because I know pharmacists only make $25 an hour which for the amount of university they do is crazy. But then I'm like people going to them there could be a real problem. |
| Theme 3: Privacy | |
| - Privacy during consultation | I don’t want to be standing at a pharmacy counter answering personal information to every man and his dog who can hear around. If I can just walk in and say can I have a look at a box of Levlen, and the one I know exactly what I want, that's okay. But if I have to go through what I go through with the GP, then I'm going to have a problem with it because I'm basically being - in a GP practice, it's just a GP and I in a room and no one else is hearing those personal details that I'm talking about. |
| - Secrecy from family | So like I said my friend, she - her mother didn't want her to take the pill, but her period pain was just so bad lying on the bed for like days and because she had to go the doctor, if she could just go to the pharmacist straight away, she wouldn't need to tell her mother. That's not a good thing to do… |
| Theme 4: Trustworthiness | |
| - GPs know more about body | I believe that, like Olga said, I completely agree with you, like they're trained for different purposes. The doctor is specifically trained to know the body and how it works and what's going on, whereas a chemist is specifically trained to know about drugs and what are the components of drugs and et cetera, et cetera. If these were to overlap, I'm not sure how it would go down. Also, even if we have a checklist, how do we know that every time a pharmacist would follow the checklist? For example, if it gets very busy, who knows, they might skip a question or two. |
| - Advice from GPs are more reliable | Just going back and they kind of match up. But going back to the advantage, disadvantage of having to go and get a prescription, is there are so many different pills to choose from that I don’t want to have to make that decision. So when a doctor just makes it for me, I'm like, "Oh yes, cool, give that one a try." And if you were going to, you know, confront the chemist or what not and there is – there may not be the same availability of, you know, 100 different options. They may only have five and those five might not work for you. |
| - GP gives better education | I think like the GP could give better education on what choices you have. I'm not sure the pharmacists would do such a thorough job. |
| - Trust in pharmacists’ knowledge and qualification | In support: And to be honest, I mean a lot of the pharmacists know more than the doctors  In opposition: I think it's, again, the education of the pharmacist, because I'm trying to remember my experience. After using it three months, I gained a lot of weight, and I stopped it because I didn't get any qualified information from the pharmacist, right? Because if the pharmacists say, "Oh, because of that you should, you know, you should use some other brands," or something like that, I would do that. Because I didn't get that information. So maybe I stop it. |
| - Ensuring safety of long term use | I completely agree with that. Like I was on the pill for 14 years and it was actually masking diabetes. So once I came off it, I just got diabetes. I was like, "What?" And they're like, "Oh, you know, you probably should have had bloods a couple years ago." Like just even something on that leaflet being like, "Hey, if you've been on this for a couple of years, maybe get your bloods checked, maybe, you know, do a repeat, maybe go back to your GP, just have like a review. Just for the long term." |
| - Turnaround rate of the pharmacists is high | If you're going to a different pharmacist, how do they know what tablets you're taking if you're just going for a quick question about a pill? |
| - Pharmacist’s’ advice might get influenced by the pharmaceutical companies | I just thought of something. One pharmacy prescribed one particular pill than another, you know what I mean, could they be influenced by the pharma companies to… |
| - Stigma from the GP | Yes, 100 percent. I mean one of my friends, her GP is heavily Catholic, and she went to get the pill and didn't know that she was heavily Catholic. And they're like, "Oh, but the Bible says this." And you know, there are some doctors that are really stepping over that line. And some even refusing to trade particular patients who want that kind of care from them. That's a problem too. |
| Theme 5: Opportunistic health screening and family issues | |
| - STIs and other reproductive health related issues might get overlooked | I would agree though with that, it's like if you did go, like if you were at the doctor's and they said they mentioned do you want to get a check-up, then you could do it right then and there, whereas if you're a pharmacy and they kind of remind you, oh yeah, maybe I should, but it's like an inconvenience to then go to the doctor. So you probably keep at the back of your head, but get distracted by life and you don’t actually get it done because it's like that added step. |
| - Getting a pap-smear test done | Oh, that's a good question. Because usually when you go for your script, the first thing they ask you is when's your last pap smear? That does prompt that question. Didn’t think about that one. |
| - Other health check-ups | I think you need to see a doctor and get your blood, your blood pressure checked and everything before you go on the pill. And your weight and everything. Because it is important. But I think if you're getting re-prescribed something and you not bring up issues, like the repeats I don’t think – I think that you should just be able to do that via telehealth consultation. |
| - Family violence might get unchecked | When I think abuse I maybe think of people who are in like abusive relationships, and then – or like maybe the age thing and at least with the GP you have to go through someone and maybe discuss things. But if you're able to just grab it and go, there's no sort of buffer for that. There's no way – I mean I'm not even sure if doctors do catch abuse in relationships that much. But I know my doctor did ask me like, do you feel – like just about the circumstances, how I felt about my relationship and stuff. Which yes, I don’t know. That's what I think of when I think of abuse in birth control. |
| - Individualised care | I would say the biggest disadvantage for me would be that like individualized care. I wouldn't receive as much. |
| Theme 6: Safety issues | |
| - Losing control over young girls | Yes, totally agree, because I'm like thinking about the thing would I want my daughter to go to the pharmacy and to get these pills? No, I don't want that. So I want to have a little bit of control over my kids till they reach a certain age, like 20, 25. And after that I'm sure that they, I'm quite confident they can, you know, by themselves. I'll let them do it. Before that I really want to, as a parent, I really want to have control over that. So yes. Over the counter, if a kid can just go and get the contraceptive, no. |
| - Easy accessibility might result into abuse of pills | I think that I agree with her contribution is like at the sense of like having, you know, the record of if a person is abusing it. So when it comes through the prescription, I think they have a record of that if the person did get the, you know, contraceptive for any reasons previously. But if they're taking from the pharmacist, if there isn't any record, because if they go to the chemist they can get it, and they, maybe next they're going to the – like a different one, like amedics or somewhere else. |
| - It might encourage teens to have sex | Does that mean they may think, oh I can have sex earlier? |
| - STIs may remain unchecked | I would agree though with that, it's like if you did go, like if you were at the doctor's and they said they mentioned do you want to get a check-up, then you could do it right then and there, whereas if you're a pharmacy and they kind of remind you, oh yeah, maybe I should, but it's like an inconvenience to then go to the doctor. So you probably keep at the back of your head, but get distracted by life and you don’t actually get it done because it's like that added step. |
| - Use of more effective contraception like LARC can go down | It might bias people towards the oral contraceptive pill when an IUD or something else may be more appropriate. So accessibility may drive people to that being the more obvious choice. Every now and then my GP asks if I want an IUD. I'm like, "Yes, that's fine." But it's been a long time. And so therefore every now and then there's a question that gets asked. So it might mean that it drives people towards that. Rightly or wrongly. I don't know. |
