# Supplementary figures and images for: A qualitative exploration of the over-the-counter availability of oral contraceptive pills in Australia
Source: PLoS One. 2024 Jun 10;19(6):e0305085. doi: 10.1371/journal.pone.0305085 (PMC11164330; doi:10.1371/journal.pone.0305085)

S3 Appendix: Mind map for over-the-counter accessibility


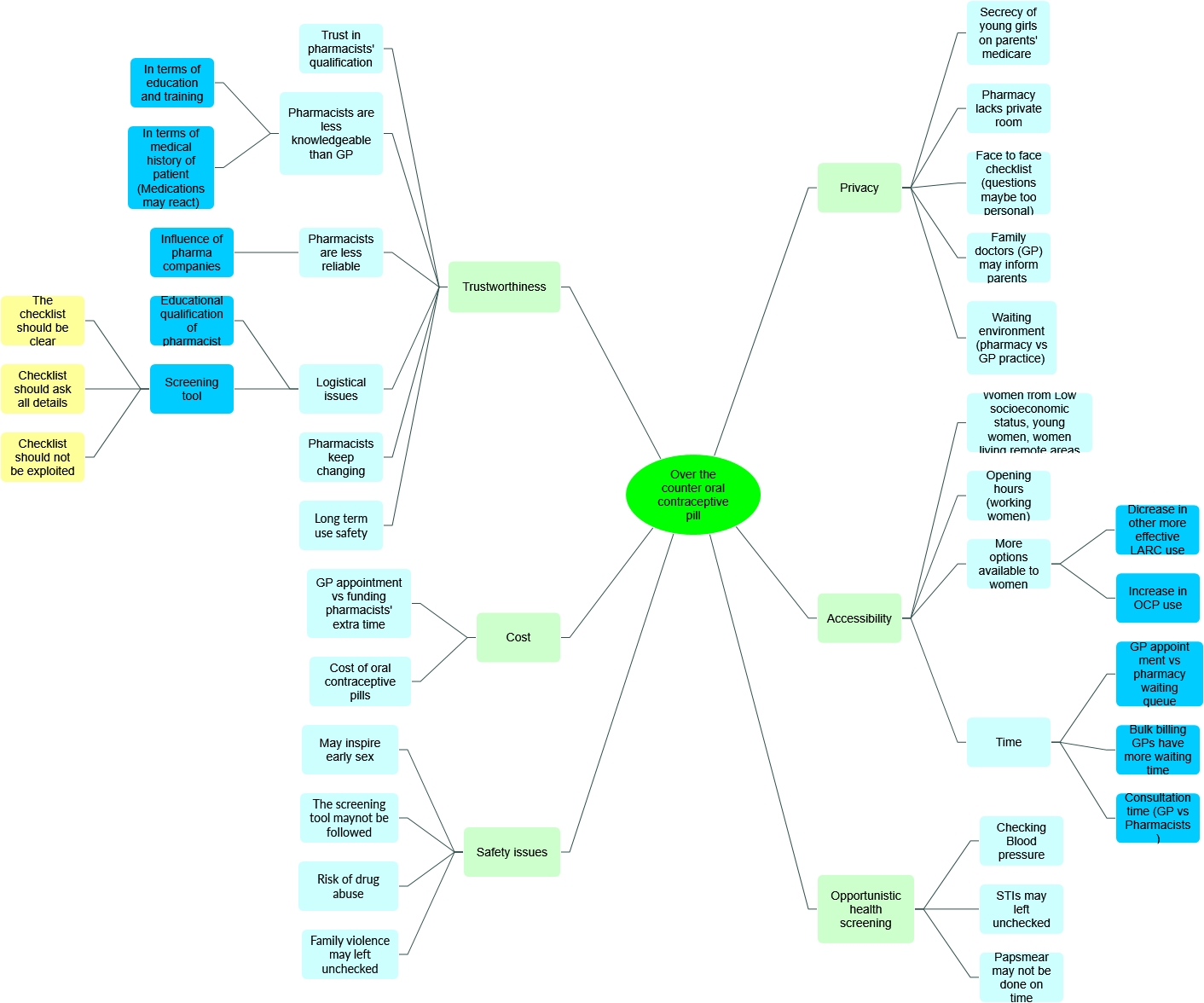

Supplement: S3 Appendix — (DOCX) [file pone.0305085.s003.docx]
